# Reporting Genetic Findings to Individual Research Participants: Guidelines From the Swiss Personalized Health Network

**DOI:** 10.3389/fgene.2020.585820

**Published:** 2020-12-11

**Authors:** Alessandro Blasimme, Caroline Brall, Effy Vayena

**Affiliations:** ^1^Health Ethics and Policy Lab, Department of Health Sciences and Technology, ETH Zürich, Zurich, Switzerland; ^2^Ethical, Legal, and Social Implications (ELSI) Advisory Group, Swiss Personalized Health Network (SPHN), Bern, Switzerland

**Keywords:** reporting, genetic research findings, return of results, ethical recommendations, expert stakeholder consultation, Switzerland

## Abstract

In 2017 the Swiss federal government established the Swiss Personalized Health Network (SPHN), a nationally coordinated data infrastructure for genetic research. The SPHN advisory group on Ethical, Legal, and Social Implications (ELSI) was tasked with the creation of a recommendation to ensure ethically responsible reporting of genetic research findings to research participants in SPHN-funded studies. Following consultations with expert stakeholders, including geneticists, pediatricians, sociologists, university hospitals directors, patient representatives, consumer protection associations, and insurers, the ELSI advisory group issued its recommendation on “Reporting actionable genetic findings to research participants” in May 2020. In this paper we outline the development of this recommendation and the provisions it contains. In particular, we discuss some of its key features, namely: (1) that participation in SPHN-funded studies as a research subject is conditional to accepting that medically relevant genetic research findings will be reported; (2) that a Multidisciplinary Expert Panel (MEP) should be created to support researchers’ decision-making processes about reporting individual genetic research findings; (3) that such Multidisciplinary Expert Panel will make case-by-case decisions about whether to allow reporting of genetic findings, instead of relying on a pre-defined list of medically relevant variants; (4) that research participants shall be informed of the need to disclose genetic mutations when applying for private insurance, which may influence individual decisions about participation in research. By providing an account of the procedural background and considerations leading to the SPHN recommendation on “Reporting actionable genetic findings to research participants,” we seek to promote a better understanding of the proposed guidance, as well as to contribute to the global dialog on the reporting of genetic research findings.

## Introduction

The Swiss Personalized Health Network (SPHN) is a Swiss federal government initiative aimed at promoting innovation in personalized medicine by making genetic and other health related data more interoperable and broadly accessible for research. A total of CHF 68 million (€ 63.5 million) was allocated to the initiative for the period of 2017–2020. During that period, a oordinated nationwide infrastructure was established to efficiently manage, exchange, and process consented health data. In doing so, SPHN has adopted a federated approach, building upon existing heterogeneous data sources and infrastructure across the country (Lawrence and Selter, 2017; Meier-Abt et al., 2018). Data sources may include clinical information systems (basic and specific data, such as imaging and lab results), clinical data registries and research data, patient and citizen self-reported user-data including data collected via mobile and wearable devices, and molecular and -omics data generated in hospitals or research facilities. Reference data sets such as environmental, geographical, and statistical data may be used as well.

Similar initiatives to SPHN have been created in recent years worldwide, for example the All of Us Research Program, the 100,000 Genome Project, and the Personal Genome Project. In establishing the SPHN, Switzerland invested in a nationwide effort to make health data “findable, accessible, interoperable, and usable for research” that is comparable to other major initiatives on the international scene (Meier-Abt et al., 2018). Hence, SPHN aims to strengthen Switzerland’s international presence in the field of personalized health-related research, leading to new scientific insights and potential benefits for patients.

The SPHN Ethical, Legal and Social Implications (ELSI) advisory group is tasked with addressing ethical, legal, and social challenges which arise related to the activities of the SPHN. Its members are experts with relevant expertise in bioethics, life sciences, law, and social sciences, as well as representatives of patient advocacy groups and key organizations in the Swiss healthcare context (such as SAMS, swissethics, National Ethics Commission). EV is the chairperson of this group. Its mandate is to advise SPHN on the development of guidelines addressing ethical challenges linked to the collection, storage, analysis and sharing of personal health-related data. In 2017, the ELSI advisory group issued ethical guidance on responsible data processing and data transfer and use, starting with The Ethical Framework for Responsible Data Processing in Personalized Health Research “The Ethical Framework for Responsible Data Processing in Personalized Health Research” (SPHN, 2018). This framework is based on four ethical principles: respect for persons, privacy, data fairness, and accountability. Each principle is followed by a set of guidelines, intended to guide the activities of researchers and institutions participating in SPHN when processing personal data or handling human biological material, in particular with a view to sharing such data and material within SPHN. This general ethical vision aims to provide a basis upon which SPHN will address all data-related matters (Meier-Abt et al., 2018).

In 2018 and 2019, SPHN’s ELSI advisory group was tasked with developing further guidance to ensure the ethically responsible handling of genetic data, this time with respect to the reporting of individual genetic research findings to participants in SPHN-funded studies. Our goal in this paper is to present SPHN’s approach in developing the guidance and to highlight its key points.

## The Process of Developing the SPHN Recommendations

The first step in the development of these recommendations was a comprehensive review of the academic literature, and of existing policy recommendations from Swiss, European, United States, and Canadian institutions concerned with reporting of individual results, such as academies of science or societies of medical genetics. This was followed by an analysis of Swiss legal provisions relevant to the reporting of research findings. The ELSI advisory group then developed a set of preliminary recommendations based on these initial findings, and guided by principles of the SPHN “Ethical Framework for Responsible Data Processing in Personalized Health Research (2018).”

The ELSI advisory group consulted with a panel of 13 experts in February 2019, to obtain diverse stakeholders perspectives on the preliminary recommendations. Experts were selected by the ELSI advisory group, based on previous consensus on stakeholder categories to include, namely: patients representatives, clinicians, geneticists, lawyers and social scientists. For each category of stakeholders ELSI advisory group members suggested names of individuals professionally active in Switzerland. The final panel included geneticists, pediatricians, sociologists, university hospital directors, patient representatives, consumer protection associations, and insurers. The experts were invited to express their views on the reporting of actionable genetic findings, providing input on the following: current reporting practices in their institutions, underlying guiding criteria and principles, advantages of making case-by-case decisions versus using defined lists, utility for individuals as well as the right not to know, and exclusion of participants who do not accept reporting of results. Stakeholder views were incorporated into the preliminary draft, which underwent further revision, before a final review by the ELSI advisory group.

## Medically Relevant Research Findings

Technological progress has steadily increased the capacity of medicine to investigate the human body. With the growth of medical imaging over the last century, for example, detailed visual representations of organs and tissues have become commonplace, at least in high-income settings. Doctors today utilize a wide array of radiologic techniques, from X-ray scans to magnetic resonance, and from ultrasound to positron emission tomography, for diagnostic and therapeutic purposes. Oftentimes these techniques may result in the unexpected discovery of a lesion, disease, or abnormality unrelated to the original purpose of the analysis. For example, an X-ray of a fracture may reveal the presence of a tumor in a nearby region. This type of finding is referred to as secondary, in contrast with primary results which are intentionally searched for. Patients generally expect doctors to report on secondary findings that arise in the course of a medical procedure, regardless of the method of diagnosis or the initial purpose of the procedure. This expectation echoes the professional commitment of doctors to “make relevant information available to patients” (American Medical Association, 1957, Principle 5). Yet communicating such results requires special attention to both the personal and medical relevance for patients. The development of clinical genetics has added another dimension to the issue of secondary findings, prompting a global debate as to whether clinically relevant genetic mutations should be communicated to patients, and under which circumstances (Ormond et al., 2019). Definitions of the terms used are provided in Table 1.

**TABLE 1:** Definitions of terms used (research context).

Term (research context)	Definition
Primary findings	Primary genetic findings are findings that are related to the primary purpose of the research protocol.
Secondary findings	Secondary genetic findings “are findings” that are outside the primary purpose of the research protocol.
Actionable findings	Primary or secondary findings that can be used to guide clinical and personal decision-making or treatment.
Medically relevant findings	Primary or secondary findings that can be used to guide medical and clinical decision-making or treatment.

Medically relevant information of potential importance to individuals is not only generated in the clinical setting. Health researchers routinely conduct medical procedures, such as imaging, as part of a study protocol, or use human tissue samples to perform molecular analyses, including genotyping. With the advent of exome sequencing and whole genome sequencing (ES/WGS) and their rapid uptake in the research context, the sheer possibility of producing secondary genetic findings has grown exponentially, posing the question of whether results (primary – related to the purpose of the study – or secondary) produced in the context of research should be communicated to participants.

Until fairly recently, the conceptual and ethical demarcations between research and medical practice stood in the way of information exchange between research teams and participants. According to this view, research activities are intended to produce generalizable knowledge for the benefit of future patients, and not to provide care – including in the form of medically relevant information or advice – to enrolled participants. From an ethical point of view, this demarcation is reflected in the need to ensure that upon giving informed consent to enrollment, study participants do not fall prey to the so-called therapeutic misconception. This notion refers to the belief on the part of research subjects that participation in a study involves receiving some form of medical care. Such a belief would signal that the person has not fully understood what study enrollment entails, an ethical precondition for participation in research. On the basis of these considerations, ethicists have long argued for a sharp demarcation between the two spheres, and for an ethical focus on preserving the participant’s autonomy. This approach has left little room to consider the existence of broader responsibilities of researchers toward participants. However, the research use of ES/WGS has changed the way we think about such obligations. In recent years, many scientific societies, medical academies, and bioethics scholars have begun to argue in favor of providing research participants with medically relevant information produced in the course of research; either primary findings (as part of a study’s scientific aim) or secondary findings (McGuire and Beskow, 2010; Anastasova et al., 2013).

Changing attitudes and expectations are evident in large-scale, publicly sponsored research programs. Precision medicine initiatives, for instance, often pride themselves on the notion of participant empowerment, promoting proactive communication to participants of both general study results and individual findings relevant to their own health (potentially including raw data upon request, thanks to data portability) (Blasimme and Vayena, 2016). Yet, not all initiatives have policies in place that determine the reporting of medically relevant findings. Empirical data confirms that public expectations for the reporting of medically relevant findings are increasing. Middleton et al., 2016 for instance report that of a sample of 6,944 individuals from 75 countries, 98% would like to receive information on findings related to preventable life-threatening conditions (2016). A study of 506 participants enrolled in the NCI’s Clinical Genetics Branch’s Familial Cancer Research Program revealed that 97% would choose to receive such results (Loud et al., 2016). In the Swiss context, willingness to be recontacted regarding medically relevant secondary findings was found to be similarly high, at 93% of 25,000 hospital patients surveyed (Bochud et al., 2017).

Furthermore, given the ever-declining costs associated with scanning the human genome for health-related variants, there is increasing consensus regarding the professional and ethical opportunity to actively check for such variants, even if they fall outside of a given research protocol (Tabor et al., 2011; Knoppers et al., 2013). Which variants researchers should search for, and under which conditions they should communicate these to patients, is still intensely debated, with multiple factors at play (Jarvik et al., 2014). An established causal correlation between a pathology and a given mutation is generally considered a necessary condition to search for that mutation and share the results with participants (Dorschner et al., 2013; Kalia et al., 2017). Inheritance patterns, the severity of the condition, the expected timeframe of disease onset, the age of the person at the time of testing, and the significance of the mutation for third parties (including genetically close relatives and future progeny) also play a role in these deliberations (Grove et al., 2014; Kalia et al., 2017). But most importantly, clinical utility – the existence of viable means to treat the condition resulting from the mutation – seems to be the factor that makes disclosure to research participants more compelling form an ethical point of view (Sharp and Foster, 2007; Bollinger et al., 2012). It is worth noting that the notion of utility can also be interpreted in a broader sense, to also include the personal utility of receiving relevant information, even in the absence of medical options to delay, prevent, or manage the pathological phenotype associated with a given genetic variant. In such cases, so the argument goes, individuals may find it useful, and indeed even crucial, to be able to arrange their life plans vis-á-vis the condition they might develop even in the absence of concrete clinical options (Bollinger et al., 2012).

The growing recognition that research participants should receive individual research findings, goes hand in hand with the acknowledgment of the right of participants to refuse such information. The widely discussed right not to know embodies the value of research participants’ autonomous choice as to whether or not they want to be informed about their genetic features. However, many argue that such a right can be overridden by the professional duty to act in the best medical interest of research participants, at least in the case of a serious but preventable condition (Bredenoord et al., 2011a). This shift of moral emphasis from autonomy toward a broader set of considerations is further evidence of the evolution of ethical thinking about the reporting of research findings over the course of the last decade.

## Ethical Principles and Legal Considerations

The responsibilities physicians have toward their patients are specified in a number of professional codes of conduct, ranging from the Hippocratic oath to the World Medical Association’s Declaration of Geneva, 1948 (originally issued in 1948, currently undergoing its 6th revision). The professional responsibilities and moral duties of medical researchers are considered to overlap with those of physicians – particularly as oftentimes those who conduct health research are also medical doctors, and those who participate in research studies are patients. However, medical research has its own professional codes of conduct, such as the World Medical Association’s Helsinki Declaration, (1964, revised for the 7th time in 2013) or CIOMS, 2016, International Ethical Guidelines for Health-related Research Involving Humans (1982, 4th version 2016).

A substantial body of academic literature in disciplines such as philosophy of medicine, ethics, and law has extensively debated the foundations of medical ethics and research ethics over the course of the last decades. In both medical ethics and research ethics there is an emphasis on avoiding harm to patients and participants, respectively, which resonates with the ancient professional pledge “primum non-nocere.” Duties of professional confidentiality have also been held in high respect since the origins of the Hippocratic tradition, and apply equally to medical practice and research. However, we have seen that the duty to promote wellbeing, enshrined in medical ethics codes since antiquity, does not clearly pertain to medical research – medical researchers do not operate under a moral obligation to actively promote the health of research subjects by means of their research activities. This assumption has been widely criticized in recent debates over reporting research findings to participants. It has been argued, for instance, that even if the principle of medical beneficence does not apply to medical researchers due to the nature and aim of research itself, medical researchers nonetheless carry a more general human responsibility toward research participants (Bombard et al., 2019). The fact that research is motivated by scientific and not by direct medical aims does not excuse researchers from other forms of moral solicitude, with respect to the people they encounter and rely on in the course of their professional activity (Knoppers et al., 2015).

Moreover, the duty to rescue those who are in need – a cornerstone of tort law as well as of medical ethics – seems apt also in the context of research. It stipulates that one should be held responsible for failing to prevent injury or death of another party, when this could be achieved without disproportionate effort or unreasonable risk (Bredenoord et al., 2011a). Failure to provide life-saving information to research participants, that is easy to generate or has been accidentally generated, constitutes a violation of such principle.

Providing general research results to participants, in addition to individual findings, is regarded as a way to acknowledge participants’ fundamental role in scientific research and to reward their participation, especially their willingness to face risk and discomfort out of an altruistic motivation to contribute to medical knowledge for the benefit of future patients (Bredenoord et al., 2011a; Wolf et al., 2012). The underlying ethical virtues of this view are reciprocity, respect, transparency, and trust.

It has furthermore been argued that researchers have ancillary care duties toward research participants because medical information that researchers gain about participants puts them in a position to affect participants’ health, either by acting or omitting to act on the basis of such information (Wolf et al., 2012). One of the most frequently cited reasons for communicating individual findings is that failure to do so shows a lack of consideration for participants as autonomous human beings, in charge of their own health-related choices (Wolf et al., 2012; Vayena and Tasioulas, 2013). Possessing knowledge about one’s health is a precondition to exercising autonomy in the sphere of personal health (Bredenoord et al., 2011b). By the same token, autonomy is invoked as a justification for the right to forego information about oneself, the so-called right not to know.

A final ethical reason to disclose individual results concerns the health of third parties. In the case of infectious disease, but also in the case of genetic mutations that could lead to pathological conditions in relatives or offspring, it seems morally sensible to consider the health of third parties on par with that of research participants. Having access to this information could prompt relevant preventive measures, further screening, or specific reproductive choices (such as pre-implantation genetic diagnosis). Therefore, the same beneficence-based justifications for offering health-related findings to individual participants, make it morally compelling to communicate findings which may affect other people. However, there is no present consensus on whether this information should also be communicated to affected third parties, such as spouses or relatives.

For the development of the SPHN guidance these principles were examined against the legal context in Switzerland. The legal landscape, on which activities related to health data in Switzerland are based, is dense. The Human Research Act, the Human Research Ordinance, the Clinical Trials Ordinance, and the Human Genetic Testing Act are of specific relevance for research using genetic data. According to these regulatory provisions, persons who take part in research or clinical trials are “entitled to be informed of results relating to their health,” and such information must be communicated in an “appropriate manner” (HRA, Art.8, 2014). Study participants also have the “right to forgo such information” (Clinical Trials Ordinance [ClinO], 2013; Human Research Act [HRA], 2018; Human Research Ordinance [HRO], 2018). The Human Genetic Testing Act (HGTA), which regulates the use of genetic results primarily in the medical context, specifies this right to self-determination by stating that “every person has the right to refuse to receive information about his or her genetic status,” indicating that individuals are free to decide if they want to undergo genetic testing, whether they want to be informed about the results, and which actions to take after receiving test results (Federal Act on Human Genetic Testing [HGTA], 2014, Art. 6). The law also requires that physicians “must immediately inform the person concerned of the test result if there is an immediate physical danger to the person, …, which could be averted” (Federal Act on Human Genetic Testing [HGTA], 2014, Art.18.2). Moreover, the HGTA stipulates that findings from genetic testing, if known to the underwriter, must be disclosed to insurance providers in the case of life insurance and invalidity insurance exceeding, respectively, an insured sum of CHF 400,000 or an annuity of CHF 40,000 (HGTA Art. 27, 2007).

## Actionable Recommendations

The SPHN guidance is structured according to general recommendations, followed by a more detailed description of what should be reported, why, and how. The recommendations promote the reporting of any genetic findings of medical relevance to participants; hence both primary and secondary genetic findings should be reported to research participants (SPHN, 2020). During the consent process, participants should be informed that medically relevant findings could be generated during the study, and should receive an explanation of their options for receipt of such findings. The potential impact of disclosing medically relevant information to third parties and on insurance applications should also be discussed. As to the question of which information to report in detail, the strongest ethical rationale favors reporting those findings for which preventive or therapeutic measures are available. However, information should also be provided about genetic variants linked to conditions that are not preventable or curable, as it could still have personal utility for the individual (i.e., it might be possible to make practical arrangements to better cope with such a condition). Furthermore, information about carrier status for a recessive Mendelian disorder should be reported to participants, as it can inform reproductive decisions. However, research participants should not receive individual information about variants of unknown relevance. Only if a participant explicitly requests information about these variants, can exceptions be made on a case-by-case basis. In conclusion, it is recommended that decisions about reporting individual findings to research participants take into account the severity of the detected condition; the existence of clinical treatment (preventive, therapeutic, or palliative); the relevance of the variant for descendants of the research participant; and the personal utility of knowing about a given mutation.

The recommendations advise researchers to relay individual genetic findings to a designated physician or medical professional, who will in turn communicate them to the research participant. Medical personnel should first confirm that the participant still wishes to be made aware of the findings, as circumstances or preferences may change over time. Validation of findings should be carried out by certified clinical-grade laboratories, and research proposal budgets must take these costs into account. Any communication of genetic findings must bear in mind the understandability of the information, psychological consequences of receiving such information, and availability of medical counseling.

The notable points of the SPHN recommendations are the following:

(1) Participation in SPHN-funded studies as a research subject is conditional to accepting that medically relevant genetic research findings will be reported;
(2) A Multidisciplinary Expert Panel (MEP) should be created to support researchers’ decision-making processes about reporting individual genetic research findings;
(3) Such Multidisciplinary Expert Panel will make case-by-case decisions about whether to allow reporting of genetic findings, instead of relying on a pre-defined list of medically relevant variants;
(4) Research participants shall be informed of the need to disclose genetic mutations when applying for private insurance, which may influence individual decisions about participation in research.

## Discussion

In general, the SPHN recommendations are in line with other international guidelines in effect today, stressing the importance of reporting scientifically valid and medically relevant results. However, guidelines vary widely over which results may, should, or must be reported to participants. Recommendations also diverge in their views about variant types, data quality, and communication of results (Thorogood et al., 2019). In this section, we discuss selected recommendations and provide more context or their justification.

### Accepting Return of Medically Relevant Genetic Research Findings Necessary for Participation in SPHN-Funded Studies

Research participants have an autonomy-based right not to be informed about individual genetic findings (primary or secondary), which is also laid out in Swiss law. Whether or not the right not to know should be upheld, however, depends on careful evaluation of its consequences. SPHN guidance suggests that it is crucial to promote the health of participants, and therefore SPHN-funded researchers should make sure medically relevant genetic findings are reported to participants. For this reason, SPHN also recommends that prospective participants who do not wish to receive individual medically relevant information not be enrolled in SPHN-funded studies. Participating in a research study is not intended to provide medical benefit to research participants, with the possible exception of some specifically designed interventional clinical trials. However, SPHN does not fund this type of studies. Therefore, it appears ethically justified and not discriminatory to exclude prospective participants who decline to receive individual genetic findings. The underlying ethical rationale is the conceptual distinction between an individual’s rights and interests. The ability to access medical treatment clearly constitutes an individual right, as it is a precondition to protect a patient’s life or bodily integrity, that is, a key component of human wellbeing. On the other hand participation in research should be seen as an interest, since – with few exceptions – no direct personal benefit generally arises from participation (especially when a participant chooses not to receive individual genetic findings). It follows that denying someone the possibility to become a research participant, does not constitute a moral violation, as there can be no such thing as a duty to include someone as a participant in any research study. In addition, withholding medically relevant information from participants may create a psychological and moral burden for researchers. SPHN hence acts according to the duty to promote individual autonomy of participants, as responsible for their health and life plans (Vayena and Tasioulas, 2013). The withholding of available information would disregard this autonomy (Blasimme et al., 2017). However, the SPHN guidance states that case-based exceptions can be made.

Other initiatives promote this choice of participants to receive results or not. The 100,000 Genomes Project, for example, operates according to an opt-in approach. In this project, participants must provide their explicit consent (opt-in) to receive the results of secondary findings, but may still join the project if they do not wish to receive this information. However, potential subjects participants are excluded from participation if they refuse the return of primary results. The All of Us Research Program also foresees that participants decide whether or not to receive individual genetic findings. Participants may set preferences for which results to receive and their preferred communication channel. The All of Us March 2018 operational protocol indicated that “consent process, policies, and procedures” which would guide the reporting of genetic results, were still to be submitted for approval (National Institutes of Health, 2018). At the time, however, they had already presented an argument against the withholding of information from research participants, regardless of potential negative impacts or uncertain significance of genetic variants. Such a stance against withholding information corresponds with the SPHN approach, which upholds the autonomy of participants to be in charge of their health and life plans, while at the same time lifting from researchers the moral burden of withholding information.

### Multidisciplinary Expert Panels to Support Decision-Making

In certain cases the decision to report individual genetic findings can be fairly complex; for example, when the medical relevance of the finding is not obvious, when the research participant is a minor or an incapacitated person, or when findings are of relevance to third parties. In such cases SPHN recommends that the research team consults a Multidisciplinary Expert Panel (MEP) within their research or clinical institution. MEPs are instituted to support decision-making as to reporting individual genetic research findings. They should be comprised of members from relevant scientific and medical specialties, patient representatives, medical geneticists, genetic counselors, experts in bioethics, and as the case may be, specialists in the disease area of interest convened on a case-by-case basis. The mandate of the MEP and its potential members should be determined prior to the beginning of the project. The panel will then be convened in cases when a decision must be made over the reporting of primary or secondary medically relevant findings to a research participant. MEPs or equivalent existing bodies are expected to base their decisions on up-to-date evidence, and consider the medical, ethical, personal, familial, psychological, and public health consequences of reporting or not reporting medically relevant research findings.

Similar recommendations worldwide have supported the establishment of expert committees to assess secondary findings. The Danish Guidelines on Genomic Research (National Committee on Health Research Ethics (Denmark), 2018) recommend that research projects establish a committee of experts to assess secondary findings. The United States National Academies of Science Guidance on “Reporting Individual-Specific Research Results to Participants” (National Academies of Science US, 2018) recommends that researchers have access to appropriate expert committees. The United States National Heart, Lung, and Blood Institute Working Group’s “Ethical and Practical Guidelines for Reporting Genetic Research Results to Study Participants” asserts that findings should be reported following review by an expert panel (see Fabsitz et al., 2010). The 100,000 Genomes Project also relies on experts from the Genomics England Clinical Interpretation Partnership, consisting of “clinical genetics, cancer genomics clinicians and scientists, research, clinical, NHSE and patient representation” (Genomics England, 2017).

The function and role of MEPs can be seen as analogous to tumor boards in the clinical setting, with experts from different specialties jointly consulting over cases. Such consultation would exceed the capacity of most traditional ethics committees. Hence MEPs are a suitable choice to address decision making in these cases.

### Case-by-Case Decisions – Instead of Following Pre-defined Lists of Genes

Instead of offering a list of genetic mutations that warrant being reported to study participants, the SPHN endorses a case-by-case approach. The primary rationale for this approach is that a list of relevant variants would evolve over time, and would thus need to be continuously updated in line with new discoveries. The American College of Medical Genetics and Genomics (ACMG) has issued a list of variants that should be actively tested and routinely reported in the clinical context (Green et al., 2013; Kalia et al., 2017). While the ACMG list can be considered a useful point of reference, it needs to be revised annually. Furthermore, such list does not take into account non-actionable variants that could still be relevant for familial, reproductive, or personal utility on the individual level. Moreover, this list has been criticized for being “arbitrary,” especially since laboratories can analyze additional genes as well, according to their own criteria (Wolf et al., 2012). Case-by-case decisions as to which findings should be reported back to the individual hence present a more reasonable approach.

### Implications for Informed Consent and Private Insurance Applications in the Swiss Context

Swiss regulations, specifically the Federal Act on Human Genetic Testing [HGTA], 2014 require that genetic mutation information obtained in the course of research be disclosed when applying for certain private insurances, either as a general rule or above a certain monetary threshold. Current or previous genetic testing results, whether obtained during medical diagnosis or participation in research, must be reported for example when seeking complementary health insurance. For life insurance or disability coverage, this requirement applies when the insured sum is more than CHF 400,000 or CHF 40,000, respectively. With an average wage of 62,500 CHF, for example, the insured sum threshold for life insurance would already nearly be reached. In Switzerland, insured sums are usually fivefold the gross income: in the case of an average wage, this would already account to 312,500 CHF (OECD Data, 2018). This implies that a fairly large proportion of individuals would be affected. The recommendation therefore points out that informed consent documents must explain the potential impact of disclosure of medically relevant information on private insurance applications in Switzerland (e.g., life insurance, complementary health insurance, or daily sickness allowances).

Bélisle-Pipon et al. (2019). report that substantial lobbying efforts have attempted to reduce the life insurance threshold, so as to allow insurers access to the genetic data of a broader array of subscribers (2019). This poses concerns about discrimination on the basis of genetic results and equity of access to private insurance. Moreover, disclosing genetic information acquired through participation to insurers could result in higher premiums or exclusion from the insurance. There is a risk that this may constitute a significant disincentive for individuals to participate in research. While implications for insurance scheme applications vary among national legislation, similar conditions apply in the United Kingdom context: Genetic information derived during the 100,000 Genomes Project, for example, also has to be disclosed (Genomics England, 2020). In order to expand research activities in Switzerland which involve genetic data, an adjustment of the policy context and a legal protection against exclusion from insurance or similar disadvantages will have to be considered (Blasimme et al., 2019).

### Need for Ethically Adequate Standards

Current trends in biomedical research indicate the increasing use of big data and the integration of genetic and genomic data in big data repositories (Vayena and Blasimme, 2018). Intense research efforts on polygenic risk scores are a paradigmatic example of how this integration is taking place in the domain of personalized medicine (Torkamani et al., 2018). The possibility of harvesting big data for diagnostic and prognostic purposes is further sustained by rapid progress in the application of artificial intelligence to the analysis of research data (Blasimme and Vayena, 2020). For these efforts to succeed, ethical, legal, and societal issues resulting from sharing increasing volumes of data among researchers must be addressed (Vayena and Blasimme, 2017; Blasimme et al., 2017). The research biobanking ecosystem must therefore prepare for the consolidation of data-driven biomedical research by adopting appropriate accountability standards (Gille et al., 2020). Ethically adequate standards on the reporting of genetic research results are a key component of accountability for personalized health research.

## Conclusion

The SPHN’s ELSI advisory group has issued recommendations on how to report findings generated in the context of genetic research. The method of consultation with expert stakeholders is particularly beneficial, as it provides the opportunity for cross-disciplinary learning with potential to enrich ethical deliberation in tangible ways. Similar examples of such an open approach to framing ethical recommendations are public hearings and workshops held by The German Ethics Council or the Nuffield Council of Bioethics, which stress inclusion of the voices of various stakeholders, including the public. In the case of the SPHN recommendations, this approach enabled an open discussion with all stakeholders, and thereby tailored an approach which specifically fits the Swiss research context supported by SPHN funds.

## Author Contributions

EV and AB designed the project. AB and CB drafted the manuscript. EV revised, edited, and commented on the manuscript. All authors read, reviewed, and approved the final manuscript.

## Conflict of Interest

The authors declare that the research was conducted in the absence of any commercial or financial relationships that could be construed as a potential conflict of interest.
